# Dietary effects of vitamin C on antioxidant capacity, intestinal microbiota and the resistance of pathogenic bacteria in cultured Silver pomfret (*Pampus argenteus*)

**DOI:** 10.1371/journal.pone.0300643

**Published:** 2024-07-02

**Authors:** Chun-Yang Guo, Ming Ding, Shun Zhang, Yi Wang, Yi-Ping Ji, Shan-Liang Xu, Ya-Jun Wang, Dan-Li Wang

**Affiliations:** 1 School of Marine Science, Ningbo University, Ningbo, China; 2 Collaborative Innovation Center for Zhejiang Marine High-efficiency and Healthy Aquaculture, Ningbo University, Ningbo, China; 3 Ningbo Hongmeng Testing Co., Ltd., Ningbo, China; 4 Ningbo Institute of Oceanography, Ningbo, Zhejiang, China; 5 Ningbo Tianbang Feed Technology Co., Ltd, Ningbo, China; Tanta University Faculty of Agriculture, EGYPT

## Abstract

As most teleosts are unable to synthesize vitamin C, supplemental diets containing vitamin C diets play a crucial role in fish health. The aim of this study was to investigate the effect of dietary vitamin C on the intestinal enzyme activity and intestinal microbiota of silver pomfre (*Pampus argenteus*). Four experimental diets were supplemented with basic diets containing 300 mg of vitamin C/kg (group tjl3), 600 mg of vitamin C/kg (group tjl6), and 1200 mg of vitamin C/kg (group tjl12), as well as vitamin C-free supplemental basic diet (group tjl0), respectively. The four diets were fed to juvenile *P*. *argenteus* (average initial weight: 4.68 ± 0.93 g) for 6 weeks. The results showed that the activity of SOD (superoxide dismutase) and CAT (catalase) increased significantly while that of MDA (malondialdehyde) decreased significantly in group tjl3 compared to vitamin group tjl0. At the genus level, groups tjl0, tjl6, and tjl12 contained the same dominant microbial community, *Stenotrophomonas*, *Photobacterium*, and *Vibrio*, whereas group tjl3 was dominated by *Stenotrophomonas*, *Delftia*, and *Bacteroides*. Among the fish fed with a basic diet containing 300 mg of vitamin C/kg, the intestines exhibited a notable abundance of probiotic bacteria, including lactic acid bacteria (*Lactobacillus*) and Bacillus. The abundance of *Aeromonas* in groups tjl3 and tjl6 was lower than that of the vitamin C-free supplemental basic diet group, whereas *Aeromonas* was not detected in group tjl12. In addition, a causative agent of the disease outbreak in cultured *P*. *argenteus*, *Photobacterium damselae* subsp. *Damselae* (*PDD*) was the dominant microbiota community in groups tjl0, tjl6 and tjl12, whereas the abundance of *PDD* in group tjl3 was the lowest among the diets. Taken together, the diets supplied with vitamin C could influence the composition microbial community of *P*. *argenteus*. The low level of vitamin C (300 mg of vitamin C/kg per basic diet) supplementation could not only improve the antioxidant capacity but also resist the invasion of pathogenic bacteria.

## Introduction

*P*. *argenteus*, a kind of migratory pelagic fish, is widely distributed in regions such as the Indian Ocean, North Sea, Persian Gulf, Oman Sea, and the coastal areas of the China Sea from the Bohai Sea to the South China Sea [1, 2]. Due to its high market demand and overfishing, its natural resources of this fish have seen a decline. Consequently, efforts towards the artificial breeding techniques of *P*. *argenteus* have been studied in both China and Kuwait since the 1980s [3]. While significant advancements have been made in the field of *P*. *argenteus* aquaculture, including the development of breeding stock, breeding and hatching, and larval rearing, there still exist certain issues that need to be addressed, such as diseases and a low immunity [4]. Furthermore, despite successfully breeding millions of juvenile *P*. *argenteus* in 2018, the problem of high mortality rates has been persisting due to diseases and the low immunity [5]. Therefore, it remains crucial to reduce mortality rates and enhance immune responses during the aquaculture of *P*. *argenteus*.

Vitamin C, also known as the ascorbic acid, plays a pivotal role as a vital water-soluble micronutrient, supporting essential physiological functions and holding the potential to bolster immune responses [6]. Because of the lack of the terminal enzyme L-gulonolactone oxidase, the majority of fish species possess a limited capacity to synthesize vitamin C, as this enzyme is responsible for the final step of vitamin C biosynthesis [7]. Vitamin C deficiency can lead to various disorders, such as an increased mortality and a suppressed immunity. Therefore, the majority of farmed fish requires a dietary vitamin C intake to maintain physiological functions and enhance immune responses [8]. Dietary vitamin C can influence the immune responses of in various cultured fish through its antioxidant capacity. Immunological indicators like SOD, CAT, and MDA offer insights into the immune responses of fish. SOD and CAT, functioning as antioxidant enzymes, are capable of neutralizing excessive harmful reactive oxygen species (ROS), thereby mitigating damage arising from lipid peroxidation [6]. MDA is produced as an end product of lipid peroxidation, which possesses strong biotoxicity, damages cell structures and functions, serving as an indicator of oxidative cell damage [6]. Therefore, evaluating the effect of vitamin C on fish antioxidant defense can involve examining the activity of SOD and CAT as well as the level of MDA. As of our knowledge cutoff date, information regarding the vitamin C requirements of *P*. *argenteus* for vitamin C is not available.

Intestinal microbiota is crucial for host fish, as it affects the feeding behavior, physiological functions, nutrient absorption, and immune responses of fish [9–11]. Diverse dietary sources can potentially impact the immune function and intestinal microbiota of fish. Numerous studies have delved into the repercussions of various diets on the composition of intestinal microbiota composition of fish species like rainbow trout (*Oncorhynchus mykiss*), crucian carp (*Carassius auratus gibelio*♀× *Cyprinus carpio*♂), Atlantic salmon (*Salmo salar*), gilthead sea bream (*Sparus aurata*), and hybrid grouper (*Epinephelus fuscoguttatus*♀×*Epinephelus lanceolatus*♂) [12–17]. Moreover, changes in the composition and diversity of the intestinal microbiota can potentially impact the hosts’ health [18]. Additionally, in the development of aquaculture production systems, the diversity of fish intestinal microbiota has garnered attention, but there is still a lack of information linking microbiomes to farm performance and fish health [19]. Therefore, understanding the impacts of vitamin C on the microbiota is crucial for comprehending the relationship between the intestinal microbiota of *P*. *argenteus* and its health. In this study, the effect of dietary vitamin C on intestinal digestive enzyme activity and intestinal microbiota of aquacultured *P*. *argenteus* is investigated in this study.

## Materials and methods

### Animals and feeding trial

*P*. *argenteus* was rare at the Xiangshan Bay, Zhejiang, China. In feeding trials, the *P*. *argenteus* was maintained with vitamin C-free supplemental basic diets and different vitamin C (Vc-2-tripolyphosphate from Ruitli Biotechnology Co., Ltd.) levels (300, 600, and 1200 mg of vitamin C/kg per basic diet) for 6 weeks. The vitamin C-free supplemental basic diet, as well as the basic diets containing 300, 600, and 1200 mg of vitamin C/kg was named as tjl0, tjl3, tjl6, and tjl2, respectively. Before the experiment, 1200 juvenile fish (average initial weight: 4.68 ± 0.93 g) were cultured in a pool of 25 m^2^, with a water depth of 1 m, which were randomly assigned to 12 pools with each pool containing 6t of sand-filtered seawater. Three replications were contained in the groups of vitamin C-free supplemental basic diet, as well as those of basic diets containing 300 mg, 600 mg, and 1200 mg of vitamin C/kg, which contained 300 individuals per pool, respectively. The basic diets contained the commercial diets (Yubao, Hayashikane Sangyo Co., Ltd.) and mackerel of *Scomberomorus niphonius*. The main raw materials of commercial diets are krill meal, fish meal, squid meal, and shrimp meal. An analysis of the commercial diet is showed in Table 1. The ratio of commercial diets and the mackerel was 2:1, respectively. The commercial diets and mackerel were mixed with water (or the water was mixed with vitamin C of different concentrations) as a sticky diet. A control diet of *P*. *argenteus* was fed with basic diets. During the feeding trials, adhesive diets were employed, with each diet freshly prepared to be fed to the fish twice a day, at 08:00 and 16:00 respectively, until reaching a point of apparent satiation. During the feeding trails, the water conditions were as follows: temperature was 23.2–31.5°C, salinity was 20–26, and the pH was at 7.8–8.1.

**Table 1:** Analysis of the commercial diet for *P*. *argenteus*.

Analyses	Composition
**Crude protein**	52%
**Crude lipid**	8%
**Crude fibre**	17%
**Crude ash**	1.5%
**Moisture**	10%
**Digestible phosphorus**	1.5%

### Sampling

At the end of feeding trials, the fish in each pool were kept not being fed for 24 h. All fish experiments were performed as recommended by the National Institutes of Health Guide for the Care and Use of Laboratory Animals. The Animal Care and Use Committee of Ningbo University approved our study protocols (#2015051701). For sampling the fish, MS-222 (100 mg/L, Tricaine methanesulfonate, Sigma-Aldrich Co. LLC.) was used to anesthetize them. Samples of lateral capsules, stomach, pyloric caeca and intestines from each treatment (three fish in each pool) were collected from each treatment (three fish in each pool) and stored at -80°C immediately. These tissues were used for analyzing the endogenous enzyme activity. Additionally, the digestive tract was separated from their abdominal cavity aseptically, and then the contents of the intestines were sucked and stored in microbes. Six fish were sampled from each pool and the intestinal content of every three of which were pooled together, therefore 2 samples were collected from each experimental group. The samples were immediately stored in liquid nitrogen. After that, a total of 8 samples, from 4 experimental diets, were kept in -80°C until analysis.

### Activity of digestive and antioxidant enzymes

The lateral capsules, stomach, pyloric caeca, and intestinal tissues were individually homogenized in an ice-cold physiological saline solution, using a volume ratio (w v−1). Subsequently, 3,000g of the homogenates were centrifuged at 4°C for 5 minutes, facilitating the separation of the supernatant, which was used for analysis of the activity of digestive and antioxidant enzymes. Total protein quantification was performed utilizing tissue homogenate and the Micro BCA Protein Assay Kit (CWBIO0) in accordance with the manufacturer’s guidelines. Three independent biological replicates were used for each analysis. After that, a specific commercial kit (Nanjing Jianchen Bioengineering Institute, Nanjing, China) was used to determine the activity of digestive enzymes such as lipase, amylase, and trypsin, as well as antioxidant enzymes such as SOD, CAT, and MDA. Lipase, amylase, trypsin, SOD, CAT, and MDA in tissues were measured with a spectrophotometer at 420, 540, 253, 550, 405, and 532nm, respectively, following the kit instructions.

### Intestin microbiota analysis

Total genome DNA of samples feed with the four diets was extracted using the CTAB/SDS method. The concentration and purity of DNA were monitored using a 1% agarose gel. Based on the concentration, DNA was diluted to 1 ng/μL using sterile water. Distinct regions of the 16S rRNA, 18S rRNA, or ITS genes, such as 16S V4, 16S V3, 16S V3-V4, 16S V4-V5, 18S V4, 18S V9, ITS1, ITS2, Arc V4, and others, were selectively amplified using specialized primers (for example, 16S V4: 515F-806R, 18S V4: 528F-706R, 18S V9: 1380F-1510R, and so on), incorporating corresponding barcodes. All PCR reactions were carried out within a reaction volume of 30 μL reaction volume, incorporating 15 μL of Phusion® high-fidelity PCR master mix from New England Biolabs, 0.2 μM of both forward and reverse primers each, and approximately 10 ng of template DNA. The thermal cycling protocol initiated with an initial denaturation step at 98°C for 1 minute, succeeding after 30 cycles. Within each cycle, denaturation was carried out at 98°C for 10 seconds, followed by annealing at 50°C for 30 seconds, and ultimately, an extension at 72°C for 30 seconds. Finally, there was a 5-minute extension step at 72°C. To detect the PCR products, an equal volume of loading buffer containing SYBR green of an equal volume was mixed with the PCR products and electrophoresed on using a 2% agarose gel. An equidensity ratio was used to mix PCR products. A GeneJETTM gel extraction kit (Thermo Scientific) was used to purify the mixed PCR products. In accordance with the manufacturer’s recommendations, sequencing libraries were generated using Thermo Scientific’s ion plus fragment library kit 48 rxns, which was assessed using a Qubit@ 2.0 fluorometer (Thermo Scientific). Lastly, 400 bp/600 bp single-end reads were produced on an Ion S5TM XL platform.

Single-end reads were allocated to samples based on their unique barcodes, which were truncated by removing the barcode and primer sequences. Quality filtering was applied to the raw reads, obtaining high-quality clean reads through the specific filtering conditions of the Cutadapt (V1.9.1, http://cutadapt.readthedocs.io/en/stable/) quality control process. The reads were compared to the reference database (Silva database, https://www.arb-silva.de/) using the UCHIME algorithm (UCHIME Algorithm, http://www.drive5.com/usearch/manual/uchime_algo.html) to detect chimera sequences. Afterwards, the chimera sequences were eliminated [20–22]. Following this process, clean reads were obtained. A sequence analysis was conducted using Uparse software (Uparse v7.0.1001, http://drive5.com/uparse/) [23]. The same operational taxonomic units (OTUs) were assigned to sequences with a similarity of greater than 97%. To facilitate further annotations, a representative sequence was selected for each OTU. Taxonomic information on each representative sequence was annotated using the Silva database (https://www.arb-silva.de/) based on the Mothur algorithm [20]. Alpha diversity was utilized to assess the intricacy of species diversity within a given sample, employing a set of six indices: observed species, Chao1, Shannon, Simpson, ACE (Abundance-based Coverage Estimator), and good coverage. All these indices in our samples were computed using QIIME (Version 1.7.0) and visualized with R software (Version 2.15.3). Community richness, evaluated through the Chao 1 estimator (link: http://www.mothur.org/wiki/Chao) and ACE estimator (link: http://www.mothur.org/wiki/Ace), along with community diversity measured by the Simpson index (link: http://www.mothur.org/wiki/Shannon), were subjected to analysis using QIIME. A functional prediction through ax4Fun harnessed the nearest neighbor approach, hinging on the minimal similarity threshold of 16S rRNA sequences. This process involved procuring prokaryotic whole-genome 16S rRNA gene sequences from the KEGG database and aligning them against the SILVA SSU Ref NR database using the BLASTN algorithm (employing a BLAST Bitscore threshold of >1500). The outcome was a correlation matrix that established connections among the sequences. The functional insights derived from prokaryotic whole genomes found in the KEGG database, annotated through UProC and PAUDA, were subsequently integrated with the SILVA database for the purpose of functional annotation within SILVA. With SILVA database sequences as references, the sequenced samples were clustered into OTUs, yielding valuable functional annotation information.

### Phylogenetic analysis

A phylogenetic analysis was conducted using the Molecular Evolutionary Genetics Analysis (MEGA) software version 6.0, involving 32 nucleotide sequences, 4 of which were obtained from 16S rRNA amplicon sequencing, compared against 28 distinct sequences of *Photobacterium* spp. retrieved from GenBank. A multiple-sequence alignment of the deduced 4 sequences of *Photobacterium* sp. was performed using Clustal W. The evolutionary history was inferred using the maximum likelihood method based on the Hasegawa-Kishino-Yano model [24]. The numerical value associated with each node signified the bootstrap probability, expressed as a percentage obtained from 1000 replicates.

### Statistical analysis

The data are presented as the mean ± SD (standard deviation). The values underwent analysis through one-way ANOVA, followed by Tukey’s test, with a significance level of *P* < 0.05 indicating a statistically significant difference.

## Results

### Effect of vitamin C supplementation on growth performance, digestive enzymes and antioxidant enzymes activities

**Fig 1:**
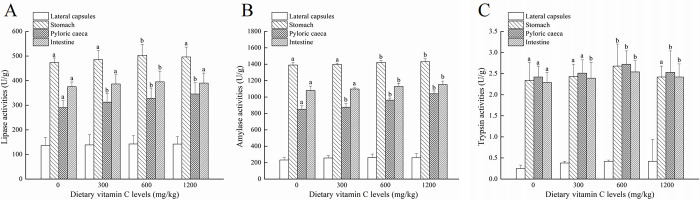
The activity of digestive enzymes at different dietary vitamin C levels. The activity of lipase (A), amylase (B) and trypsin (C) in lateral capsules, stomach, pyloric caeca and intestine tissues was detected. Superscript letters indicate one-way ANOVA, followed by Tukey’ s test (*P* < 0.05).

**Fig 2:**
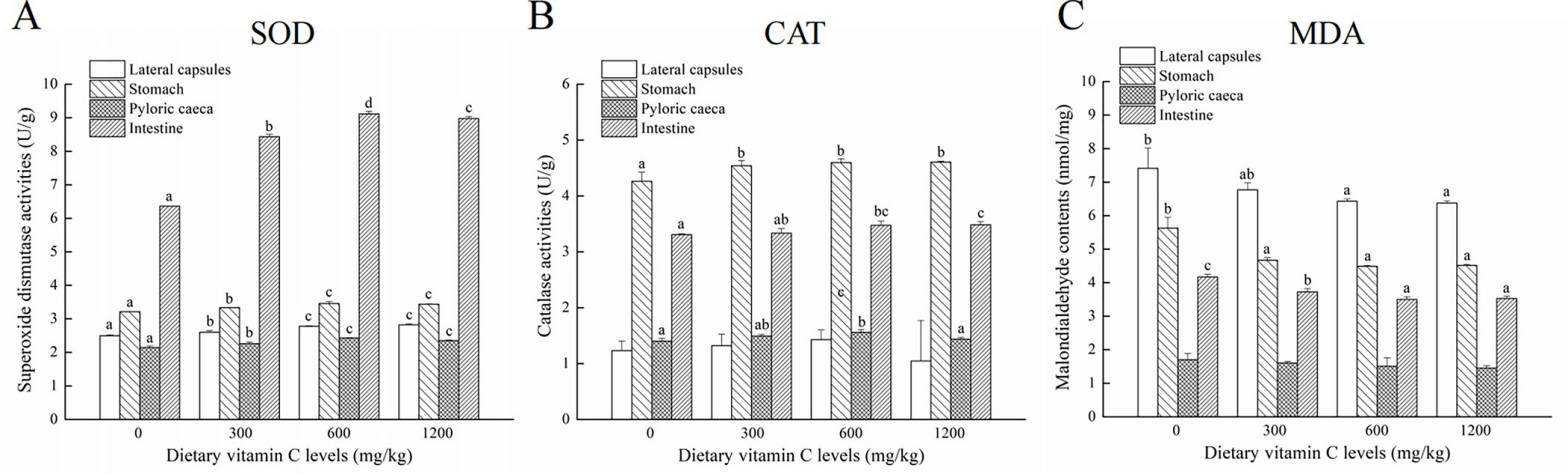
The activity of antioxidant enzymes at different dietary vitamin C levels. The activity of superoxide dismutase (A), catalase (B) and malondialdehyde (C) in lateral capsules, stomach, pyloric caeca and intestine tissues was detected. SOD, superoxide dismutase; CAT, catalase; MDA, malondialdehyde. Superscript letters indicate one-way ANOVA, followed by Tukey’ s test (*P* < 0.05).

After feeding diets with different contents of vitamin C for 6 weeks, the final body weight (FBW) was significantly affected (*P*<0.05) (Table 2). However, the survival rage (SR), weight gain rate (WGR), and specific growth rage (SGR) in different diets containing vitamin C were no significance. The effect of diets containing vitamin C diet on lipase, amylase and trypsin activity is showed in Fig 1. It was observed that the lipase, amylase, and trypsin activity increased significantly with a dietary vitamin C supply of 600 mg/kg per basic diet in stomach. For pyloric caeca, the lipase and amylase activity increased significantly with a dietary vitamin C supply of 300 mg/kg per basic diet, however, trypsin activity increased significantly with a dietary vitamin C supply of 600 mg/kg per basic diet. In intestines, the lipase and amylase activity increased significantly when dietary vitamin C supply of 300 mg/kg per basic diet, and the activity of trypsin increased in basic diets containing 300 mg of vitamin C/kg. In addition, dietary vitamin C supplementation significantly affected SOD, CAT, and MDA activity (Fig 2). The SOD activity in four different tissues increased significantly when dietary vitamin C supply of 300 mg/kg per basic diet compare to the vitamin C-free supplemental basic diets (Fig 2A). For the CAT activity, there was no difference among different diets in lateral capsules whereas increased significantly with a dietary vitamin C supply of 300 mg/kg per basic diet in stomach, pyloric caeca, and intestines (Fig 2B). Moreover, the MDA activity in lateral capsules, stomach, and intestines decreased significantly in basic diet group with 300 mg of vitamin C/kg compared to that of vitamin C-free supplemental basic diet (Fig 2C).

**Table 2:** Effects of vitamin C at different levels on the growth performance of *P*. *argenteus*.

Parameters	tjl0 diet	tjl3 diet	tjl6 diet	tjl12 diet
**IBW (g)**	4.65 ± 1.08	4.55 ± 1.04	4.80 ± 0.90	4.73 ± 0.60
**FBW (g)**	11.16 ± 0.69^b^	13.99 ± 0.74^a^	12.17 ± 0.42^ab^	11.94 ± 1.07^ab^
**SR (%)**	88.00 ± 5.10	90.33 ± 6.16	89.44 ± 3.68	88.67 ± 2.16
**WGR (%)**	141.60 ± 19.95	211.58 ± 44.29	154.02 ± 16.46	151.95 ± 17.14
**SGR (%/d)**	2.09 ± 0.20	2.68 ± 0.33	2.21 ± 0.15	2.19 ± 0.16

IBW, initial body weight; FBW, final body weight; SR (survival rate, %) = 100 × (number of survival/total number); WGR (weight gain rate, %) = 100 × [(FBW-IBW) / IBW]; SGR (specific growth rate, %/d) = 100 × {ln [FBW (g)]—ln [IBW (g)]}/42(d); tjl0 diet, vitamin C-free supplemental basic diet; tjl3 diet, group of basic diet containing of 300 mg vitamin C/kg; tjl6 diet, group of basic diet containing of 600 mg vitamin C/kg, tjl12, group of basic diet containing of 1200 mg vitamin C/kg; Values are means ± SD; Superscript letters indicate one-way ANOVA, followed by Tukey’ s test (*P* < 0.05).

### Basic features and diversity analysis

The 16S rRNA pyrosequencing process yielded a total of 645,550 raw reads. Following the merging steps, a sum of 606,532 tags were successfully extracted from the samples tjl0, tjl3, tjl6, and tjl12. The clean reads were clustered into 351, 496, 490, and 310 OTUs a sequence similarity of over 97% sequence similarity from groups tjl0, tjl3, tjl6, and tjl12, respectively (Table 3). Group tjl12 had the smallest OTU number. The analysis of rarefaction curves unveiled consistent patterns of microbial diversity across all samples, with the curves approaching a saturation plateau (S1 Fig). Group tjl3 had the largest number of OTUs and Shannon and the group tjl6 had the largest number of Chao1 and ACE. The tjl12 group had the lowest number of OTUs, Chao1, ACE, and Shannon. These data suggested that group tjl12 had the lowest diversity and richness of microbiota in the gut among different levels of vitamin C supplements of different levels, however, groups tjl3 and tjl6 had a higher diversity and richness than group tjl0.

**Table 3:** Alpha diversity analysis of the intestinal bacteria of *P*. *argenteus* with different vitamin C supplementation.

Feed group	OTUs	Chao1	ACE	Shannon	ECS
**tjl0 diet**	351	392.396	398.136	3.825	0.998
**tjl3 diet**	496	507.334	507.334	4.760	0.998
**tjl6 diet**	490	515.806	515.806	4.327	0.999
**tjl12 diet**	310	372.979	372.979	2.117	0.998

tjl0 diet, vitamin C-free supplemental basic diet; tjl3 diet, group of basic diet containing 300 mg of vitamin C/kg; tjl6 diet, group of basic diet containing 600 mg of vitamin C/kg; tjl12, group of basic diet containing 1200 mg of vitamin C/kg; OTUs, Operational Taxonomic Units; ACE, Abundance-based Coverage Estimator; ECS, equal correlated score.

### The dominant intestinal microbiota

**Fig 3:**
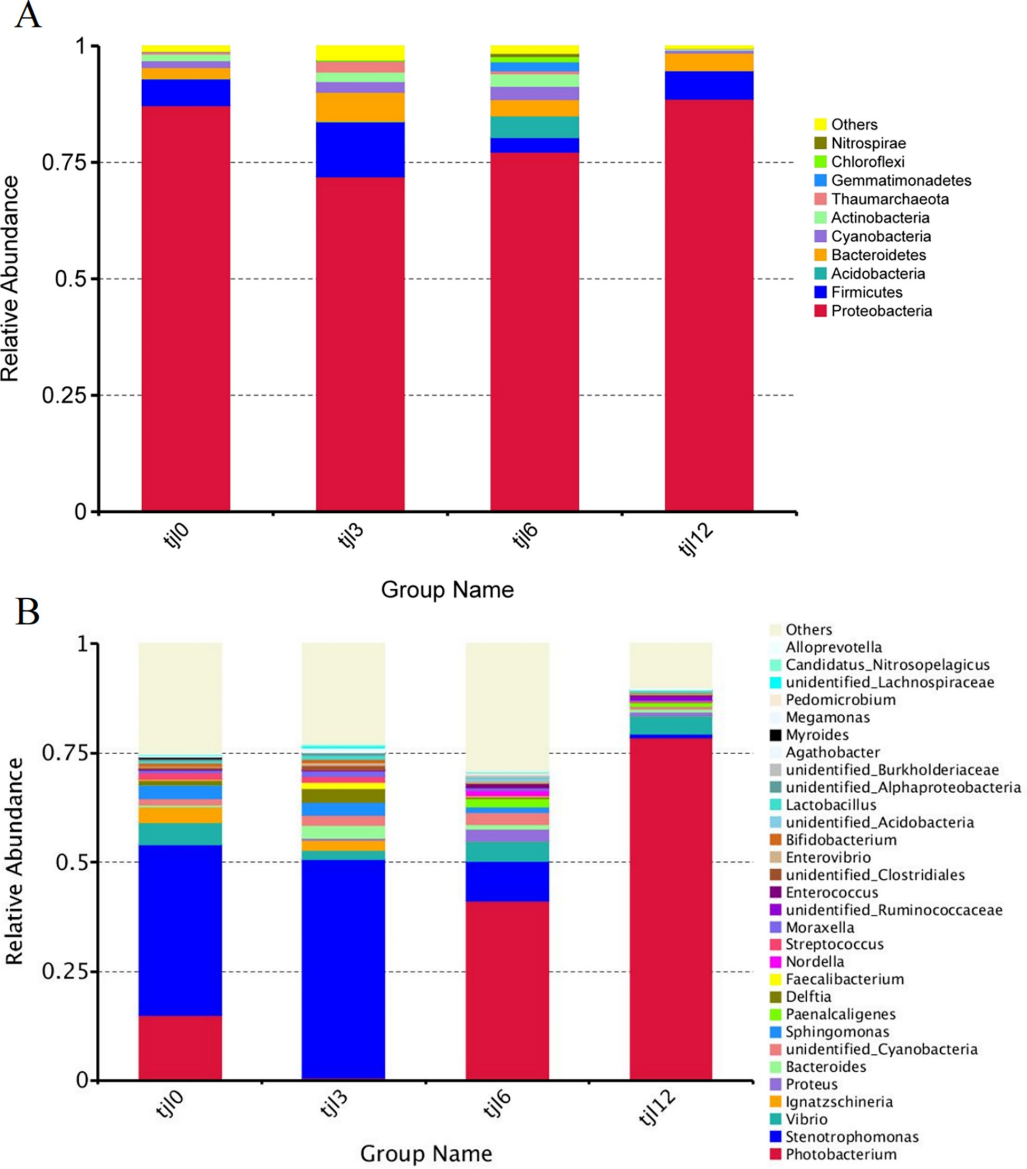
The relative abundance of intestinal microbiota at different dietary vitamin C levels. (A) The top 10 most abundant phyla in all diets; (B) The top 30 most abundant genus in all diets. Tjl0, vitamin C-free supplemental basic diet group; tjl3, group of basic diet containing 300 mg of vitamin C/kg; tjl6, group of basic diet containing of 600 mg vitamin C/kg; tjl12, group of basic diet containing 1200 mg of vitamin C/kg.

Proteobacteria represented the most relatively abundant phylum in groups tjl0 (87.24%), tjl3 (71.94%), tjl6 (77.26%), and tjl12 group (88.62%) (Fig 3A). The top 10 abundant phyla detected in group tjl0 were Proteobacteria (87.24%), Firmicutes (5.70%), Bacteroidetes (2.36%), Cyanobacteria (1.48%), Actinobacteria (1.42%), others (1.09%), Thaumarchaeota (0.53%), Acidobacteria (0.10%), Chloroflexi (0.05%), Gemmatimonadetes (0.02%), and Nitrospirae (0.01%). In group tjl6, the intestinal microbiota was dominated by Proteobacteria (77.26%), Acidobacteria (4.66%), Bacteroidetes (3.49%), and Firmicutes (3.11%). The top 3 abundant phyla in groups tjl3 and tjl12 groups were the same as those in the vitamin C-free supplemental basic diet group. In addition, the top 10 abundant phyla detected in group tjl12 were Proteobacteria (88.62%), Firmicutes (6.11%), Bacteroidetes (3.76%), Cyanobacteria (0.60%), Actinobacteria (0.29%), Thaumarchaeota (0.12%), Acidobacteria (0.002%), Chloroflexi (0.00008%), Gemmatimonadetes (0.00003%), Nitrospirae (0.00003%), and others (0.47%). In diets with different levels of vitamin C diets, the composition of the top 10 most abundant OTUs in group tjl12 at the phylum level was the closest to that of group tjl0 group.

The top 30 most abundant OTUs in all diets were targeted at the genus level (Fig 3B). OTUs in group tjl0 were assigned to 28 genera, with the least abundant OTUs being assigned to *Nordella* with a relative abundance of 0.0015%. OTUs in groups tjl3, tjl6, and tjl12 groups were assigned to 27, 29 and 28 genera, respectively. The fish intestinal microbiota community of group tjl0 was dominated by *Stenotrophomonas* (relative abundance of 39.12%), *Photobacterium* (15.03%), and *Vibrio* (5.05%), while *Ignatzschineria* (3.42%), *Sphingomonas* (3.13%), and unidentified *Cyanobacteria* (1.48%) were subdominants (Fig 3B). Also, both groups tjl6 and tjl12 had the same dominated intestinal microbiota community. Group tjl6 was dominated by *Photobacterium* (41.20%), *Stenotrophomonas* (9.13%), and *Vibrio* (4.51%), while group tjl12 was dominated by *Photobacterium* (78.45%), *Vibrio* (4.16%), and *Stenotrophomonas* (0.98%). However, the dominated intestinal microbiota community in group tjl3 was *Stenotrophomonas* (50.17%), *Delftia* (3.07%), and *Bacteroides* (3.02%).

### Microbiota differences among different diets

**Fig 4:**
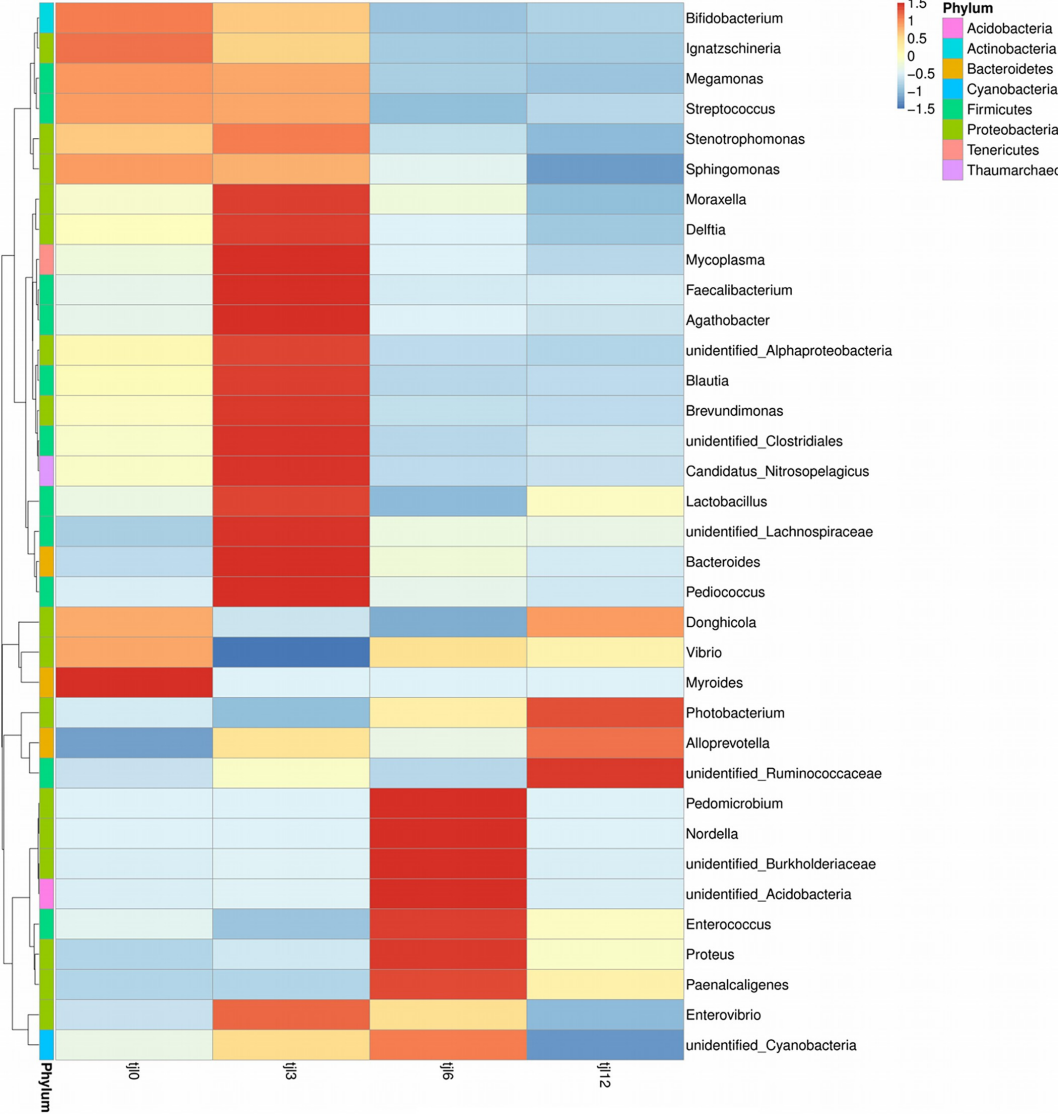
Taxonomic distribution at the phylum level with different dietary vitamin C levels. Tjl0, group of vitamin C-free supplemental basic diet; tjl3, group of basic diet containing of 300 mg vitamin C/kg; tjl6, group of basic diet containing of 600 mg vitamin C/kg; tjl12, group of basic diet containing of 1200 mg vitamin C/kg.

**Fig 5:**
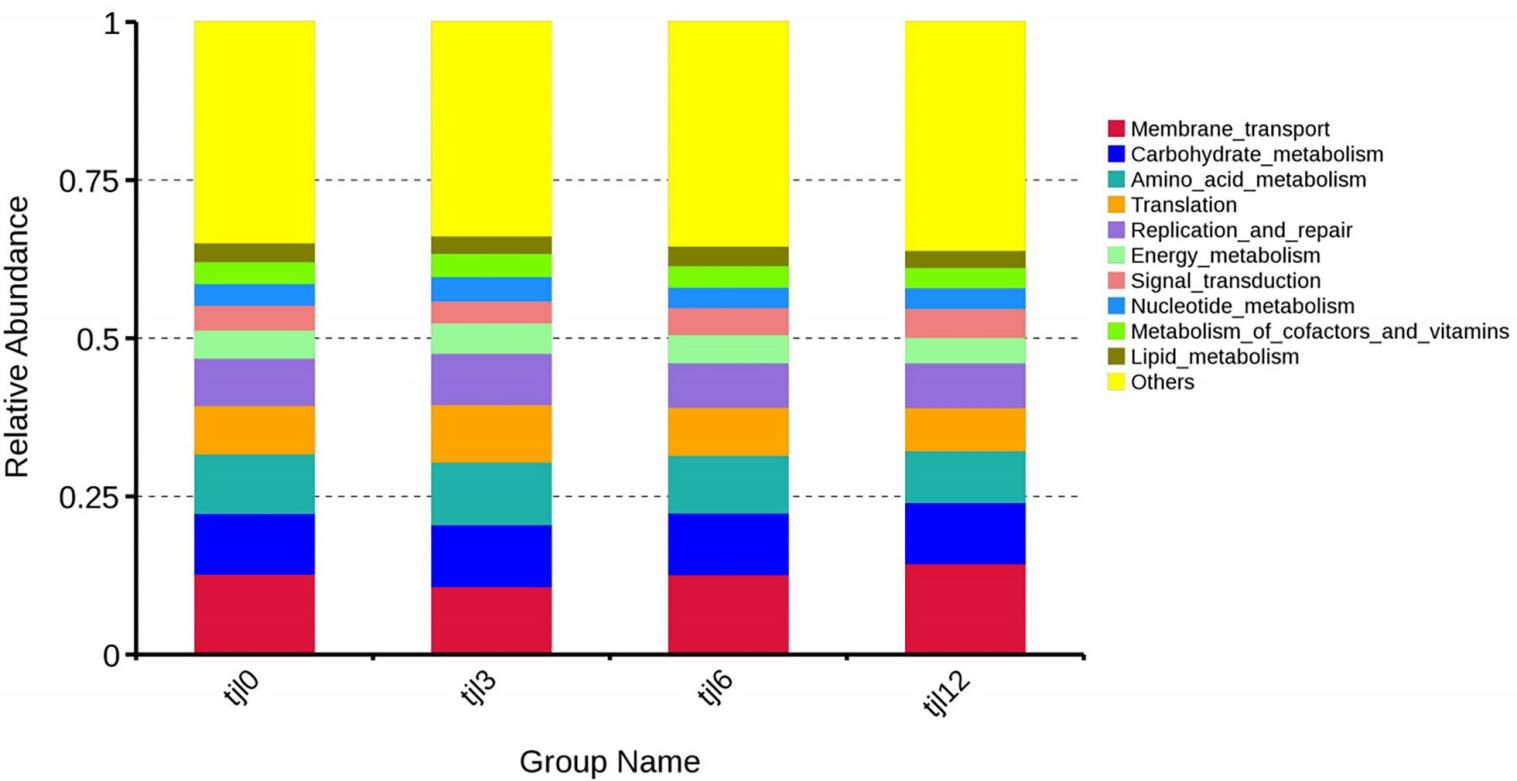
The function prediction heatmap of Tax4Fun at different dietary vitamin C levels. Tjl0, group of vitamin C-free supplemental basic diet; tjl3, group of basic diet containing of 300 mg vitamin C/kg; tjl6, group of basic diet containing of 600 mg vitamin C/kg; tjl12, group of basic diet containing of 1200 mg vitamin C/kg.

Taxonomic distribution of the OTUs in the supplement of *P*. *argenteus* at different vitamin C levels is shown in Fig 4. The low similarity was observed between group tjl0 and group tjl12. The common gut bacteria of marine fish include *Vibrio*, *Bacteroides*, *Aeromonas*, *Flavobacterium*, *Pseudomonas*, and *Acinetobacter*, which were found among the different diets. At the genus level, *Vibrio* was the dominant microbiota community of groups tjl0, tjl6 and tjl12. The most relative abundance was shown in diets of group tjl0 (5.05%) and the least was shown in group tjl3 diet (2.00%) (Fig 4). *Bacteroides* was also found in the Fig 5, however, the most relative abundance was shown in group tjl3 group (3.02%) and the least was shown in group tjl0 group (0.37%). With the increasing vitamin C supplement, *Lactococcus* and *Aeromonas* decreased, even though *Aeromonas* could not be detected in group tjl12. Even though *Flavobacterium*, *Pseudomonas*, and *Acinetobacter* were not found among the top 30 most abundant OTUs in all diets, there were little among different diets. In addition, the relative abundance of the genus *Lactobacillus* was the most in group tjl3 (0.91%) and the least in group tjl6 (0.22%) (Fig 4).

### Functional prediction of the intestinal microbiota

**Fig 6:**
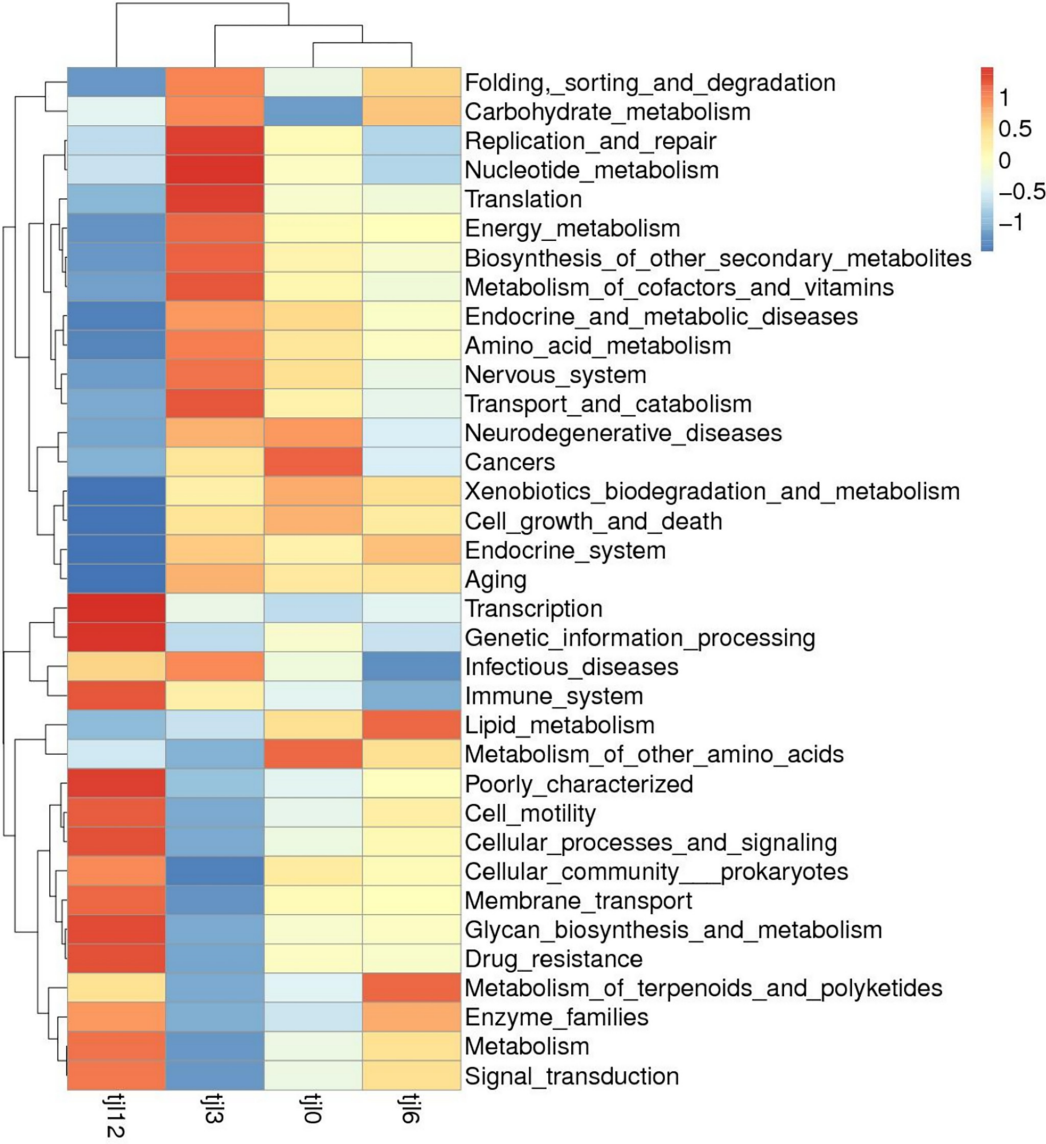
KEGG level 2 analysis at different dietary vitamin C levels. Tjl0, group of vitamin C-free supplemental basic diet group; tjl3, group of basic diet containing of 300 mg vitamin C/kg; tjl6, group of basic diet containing of 600 mg vitamin C/kg; tjl12, group of basic diet containing of 1200 mg vitamin C/kg.

Alterations in the potential functions of the intestinal microbiota in *P*. *argenteus* were investigated by employing Tax4Fun to predict metagenomes. The functional predictions unveiled an enrichment of functions linked to diverse processes including membrane transport, amino acid metabolism, carbohydrate metabolism, translation, replication and repair, energy metabolism, signal transduction, nucleotide metabolism, metabolism of cofactors and vitamins, as well as lipid metabolism (Fig 5). In a KEGG level 2 analysis, group tjl12 was the most different from group tjl0. Metabolism of cofactors and vitamins as well as infectious diseases were enriched in group tjl3 among the different diets. Immune system and enzyme families were enriched in group tjl12 (Fig 6).

### Potential probiotics and pathogens

It was found that probiotics used for fish culture, such as *Lactobacillus*, *Bacillus*, and *Lactococcus*, were also present in *P*. *argenteus*’s gut microbiota. *Lactobacillus* and *Bacillus* had the most relative abundance in group tjl3. However, the relative abundance of *Lactobacillus* in group tjl6 was less than that in group tjl0. Both groups had a lower relative abundance of *Bacillus* than group tjl0. With an increasing vitamin C supplement, *Lactococcus* and *Aeromonas* decreased, even though *Aeromonas* could not be detected in group tjl12.

**Fig 7:**
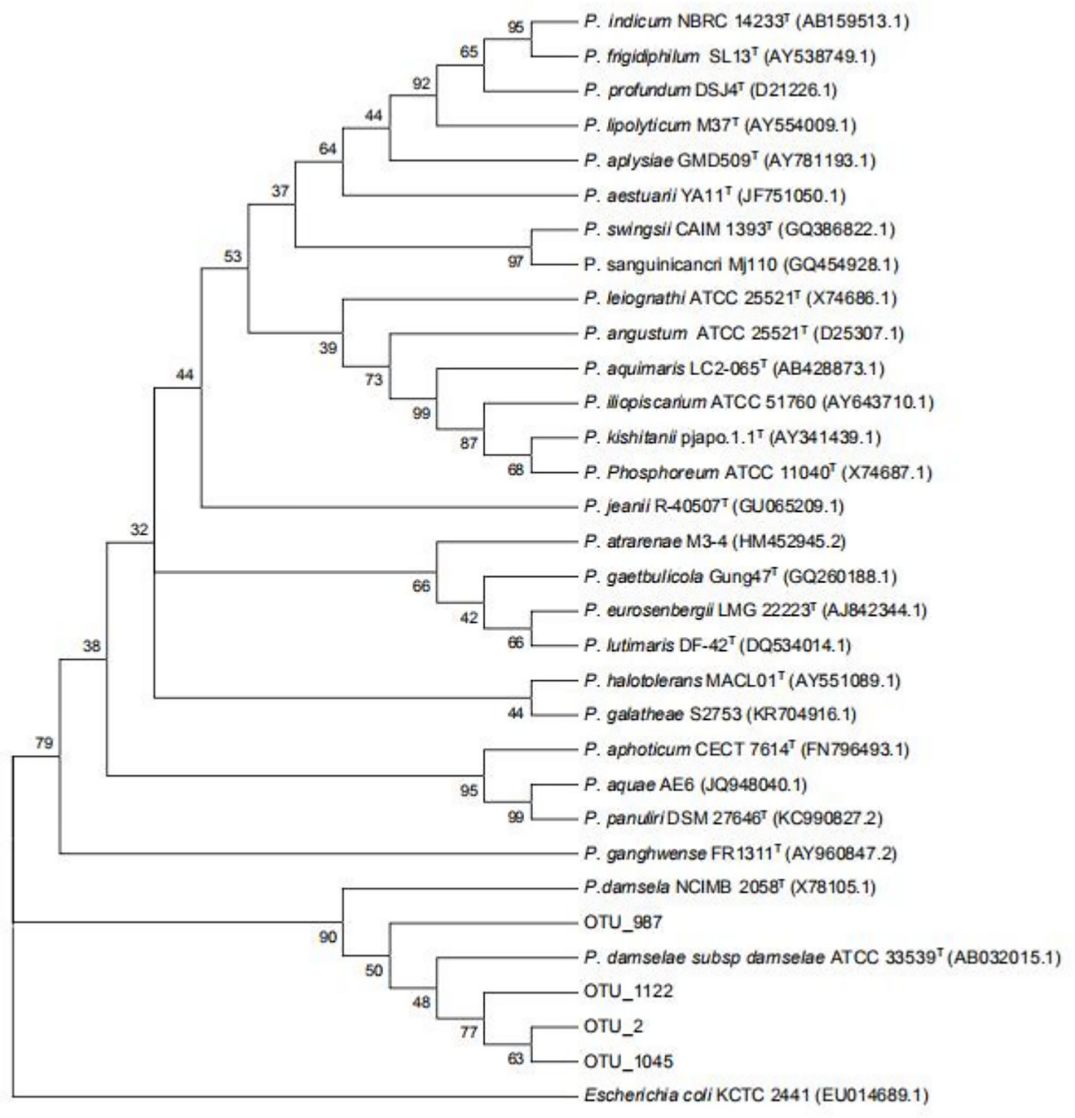
The 16S neighbor-joining phylogenetic tree of *Photobacterium* spp. The sequences of OTU 2, OTU 987, OTU 1045, and OTU 1122 were from the results of 16S rRNA sequencing.

As shown in Fig 3A, Proteobacteria represents the most relatively abundant phylum among all diets. At the genus level, *Photobacterium* and *Vibrio* were the dominated intestinal microbiota community in groups tjl0, tjl6, and tjl12 group except group tjl3 (Fig 3B). In this study, we focused on *Photobacterium damselae* subsp. *damselae* (*PDD*), which was pathogenic for aquacultured *P*. *argenteus*. Notably, only four *Photobacterium* OTUs (OTU 2, OTU 987, OTU 1045, and OUT 1122) were detected in the samples. The neighbor-joining phylogenetic tree based on the 16S rRNA sequences illustrated a closely-knit cluster formed by the four OTUs in conjunction with *PDD*, which stood apart from other clades representing various species of *Photobacterium* (Fig 7), suggesting that the pathogenic bacterium *PDD* was abundant in the intestinal microbiota of *P*. *argenteus* when not supplied or supplied with high-level vitamin C diets.

## Discussion

Digestive enzymes that can be regulated through nutritional management are able to reflect the digestive processes and nutritional status of fish [25, 26]. In this study, the lipase, amylase, and trypsin activity increased in different tissues with the dietary supply of vitamin C level at a certain concentration level. In juvenile jian carp (*Cyprinus carpio*), lipase activity was increased with an increasing vitamin C level of up to 45.1 mg/kg [27]. However, in juvenile discus fish (*Symphysodon haraldi*), lipase activity was not affected by dietary vitamin C [28]. Moreover, vitamin-C-supplemented diets improved the digestive enzyme activity of juvenile discus fish [28], Nile tilapia (*Oreochromis niloticus*) [29], and freshwater prawn (*Macrobrachium malcolmsonii*) [30]. Therefore, dietary vitamin C can improve the digestive enzyme activity of *P*. *argenteus*, thereby enhancing feed utilization. Moreover, in the present study, the activity of SOD and CAT increased significantly while that of MDA decreased significantly with a dietary vitamin C supply of 300 mg/kg per basic diet compared to the group of vitamin C-free supplemental basic diet in all tissues. However, higher levels of dietary vitamin C did not improve antioxidant capacity (1200 mg of vitamin C/kg per basic diet). In other aquatic animal species, Siberian sturgeon (*Acipenser baerii*) and yellow catfish (*Pelteobagrus fulvidraco*) responded positively to dietary vitamin C [31]. Moreover, higher dietary vitamin C levels also failed to enhance the antioxidant capacity of channel catfish (*Ictalurus punctatus*) [32] and juvenile cobia (*Rachycentron canadum*) [33]. Therefore, a vitamin C content of 300 mg/kg in the basic diets was able to improve the antioxidant capacity of *P*. *argenteus* in this study.

The microbiota community in the fish intestines is highly responsive to dietary changes [34–36]. An increasing number of studies have indicated that various dietary sources can impact the intestinal microbial community and subsequently influence the hosts’ intestinal health [12, 35, 37, 38]. The composition of diet triggers modifications in the community of intestinal microbiota within fish, resulting in consequential shifts in the hosts’ biology, subsequently exerting an impact on the metabolism and population dynamics of crucial symbiotic species [38]. Thus, in the present study, we examined the intestinal microbiota profile of the *P*. *argenteus* fed at different vitamin C levels. Research conducted previously has demonstrated that within the intestinal tracts of fish, the predominant phyla include Proteobacteria, Firmicutes, and Bacteroidetes [12, 39–41]. In this study, the group of vitamin C-free supplemental basic diet group was dominated by Proteobacteria, Firmicutes, and Bacteroidetes at the phylum level. This observation was consistent with the findings of previous studies, such as cultured sea bass, rainbow trout, and carp [12, 39–41]. Also, the same dominant intestinal microbial community as that in the of the vitamin C-free supplemental basic diet was found when supplied with 300 or 1200 mg vitamin C/kg per basic diet. The presence of these phyla in the intestine microbiota of *P*. *argenteus* suggested that they might perform various functions in the intestine ecosystem. However, when 600 mg of vitamin C/kg per basic diet was supplied, the intestinal microbial community was dominated by Proteobacteria, Firmicutes, and Acidobacteria, which indicated that 600 mg of vitamin C/kg per basic diet might influence the intestinal microbial community of *P*. *argenteus*. At the genus level, the group of vitamin C-free supplemental basic diet, as well as 600 and 1200 mg vitamin C/kg per basic diet had the same dominant microbial community, *Stenotrophomonas*, *Photobacterium*, and *Vibrio*, whereas 300 mg of vitamin C/kg per basic diet was dominated by *Stenotrophomonas*, *Delftia*, and *Bacteroides*. Nevertheless, in marine fish intestines, the prevailing microbial entities such as *Vibrio*, *Bacteroides*, *Aeromonas*, *Cytophaga*, *Acinetobacter*, *Alteromonas*, and *Pseudomonas* were consistently identified as the dominant constituents [35, 42–44]. This suggested that *P*. *argenteus* might have the different microbial community compositions at the genus level among the most marine fish. Taken together, diets supplied with vitamin C could influence the composition microbial community of *P*. *argenteus*. Also, previous research has demonstrated that diets can shape the composition of intestinal microbial communities in various fish species, including rainbow trout, sea bream, Arctic charr (*Salvelinus alpinus*), yellowtail kingfish (*Serio lalalandi*), channel catfish (*Ictalurus punctatus*), and field eel (*Monopterus albus*) [12].

Studies have confirmed the significant roles that intestinal microbiota and their metabolites play in digestive function, immune responses, and disease resistance [45–47]. Probiotic bacteria have the capacity to produce one or more active antimicrobial metabolites and peptides, which play a role in modulating the hosts’ immune system and promoting intestinal health [48, 49]. Numerous advantageous species collaborate to uphold the equilibrium of the intestinal microbiota by engaging in nutrient competition, product inhibition, and various other interactions. Bacillus primarily thrives in feed compositions, whereas Lactobacillus and Lactococcus are widely acknowledged as probiotics within the realm of aquaculture [50–53]. The functional effect of Lactobacillus on fish intestines is unknown, but they might protect fish from pathogens and enhance the immune system, which are often considered as candidates for probiotics [54]. Our investigation revealed that within the intestines of fish fed with basic diets containing 300 mg of vitamin C per kilogram, probiotic bacteria like lactic acid bacteria (*Lactobacillus*) and *Bacillus* exhibited the highest level of abundance. Although the actic acid bacterium, *Lactococcus*, was the most abundant in the group of vitamin C-free supplemental basic diet group, it was more abundant in the group of basic diet containing 300 mg of vitamin C/kg than that of 600 and 1200 mg vitamin C/kg per basic diet, suggesting that 300 mg of vitamin C/kg per basic diet could resist the invasion of pathogenic bacteria compared to other diets.

Pathogenic bacteria found within the intestines, such as *Aeromonas* and *Vibrio*, have the potential to heighten the susceptibility to infections caused by pathogens [55]. In the present study, the abundance of *Aeromonas* in groups with 300 and 600 mg of vitamin C/kg per basic diet was lower than that of the group of vitamin C-free supplemental basic diet, whereas *Aeromonas* was not detected in the group of 1200 mg of vitamin C/kg per basic diet. Furthermore, *Vibrio* is frequently regarded as the quintessential bacterium representative of vibriosis within the domain of marine aquaculture [56, 57]. Within the context of marine fish aquaculture, specific strains within the Pseudomonas genus are recognized as pathogenic bacteria, posing a threat to species such as turbot and cod [58–60]. However, although *Vibrio* was the main genus, the representative pathogen species (*V*. *anguillarum*, *V*. *harveyi*, *V*. *alginolyticus*, and *V*. *parahaemolyticus*) were not found in this study [61–64]. Interestingly, we found that not only the *Photobacterium* was the dominant microbiota community in the group of vitamin C-free supplemental basic diet or that of 600 and 1200 mg of vitamin C/kg per basic diet, but also consisted of *PDD*, potentially leading to the emergence of disease-outbreak-causing agents in aquacultured *P*. *argenteus* [4]. In addition, the abundance of *Photobacterium* in the group of 300 mg of vitamin C/kg per basic diet was the lowest among the groups. The results suggested that with a diet supply of a low vitamin C level, the abundance of *Photobacterium* in fish intestinal microbiota decreased. *PDD* is an autochthonous inhabitant of aquatic ecosystems that triggers vibriosis in a variety of marine animals, including molluscs, crustaceans, fish and even humans [4, 65–68]. *PDD* is considered a primary pathogen in several species of both wild and cultured fish, causing wound infections and hemorrhagic septicemia [69]. However, up until now, we only know that pathogenic *Photobacterium* species might possess certain virulence factors that allow them to evade the hosts’ defense system, spread within the hosts’, and ultimately lead to their demise [69]. Fish intestines often contain a variety of bacteria with the ability to inhibit pathogens [70–72]. Previous studies have indicated that the proportion of antagonistic bacteria in the intestines of Senegalese sole (*Solea senegalensis*) increases once the larvae start feeding. A majority of the intestinal microbiota showed antagonistic activity towards Photobacterium damselae after six weeks [73]. Furthermore, bacteria with broad-spectrum inhibitory activity against various pathogens were also found in the intestines of fish. In summary, further research is needed to enhance our understanding of the relationship between PDD and low vitamin C levels.

## Conclusion

In this study, we investigated the effect of dietary vitamin C on intestinal enzyme activity and intestinal microbiota of *P*. *argenteus*. The study found that dietary vitamin C could improve the digestive enzymes activity and antioxidant capacity of *P*. *argenteus*. Diets supplied with vitamin C could influence the composition microbial community of *P*. *argenteus*, and a low level vitamin C supplementation level (300 mg of vitamin C/kg per basic diet) supplementation could resist the invasion of pathogenic bacteria compared to other diets.

## Supporting information

S1 Fig The rarefaction curve of different dietary vitamin C levels. Tjl0, vitamin C-free supplemental basic diet group; tjl3, 300 mg vitamin C/kg basic diet group; tjl6, 600 mg vitamin C/kg basic diet group; tjl12, 1200 mg vitamin C/kg basic diet group.
(PDF)

S1 Data (ZIP)

S2 Data (ZIP)

S3 Data (ZIP)

S4 Data (ZIP)
