# Supplementary figures and images for: Dietary effects of vitamin C on antioxidant capacity, intestinal microbiota and the resistance of pathogenic bacteria in cultured Silver pomfret (Pampus argenteus)
Source: PLoS One. 2024 Jul 2;19(7):e0300643. doi: 10.1371/journal.pone.0300643 (PMC11218981; doi:10.1371/journal.pone.0300643)

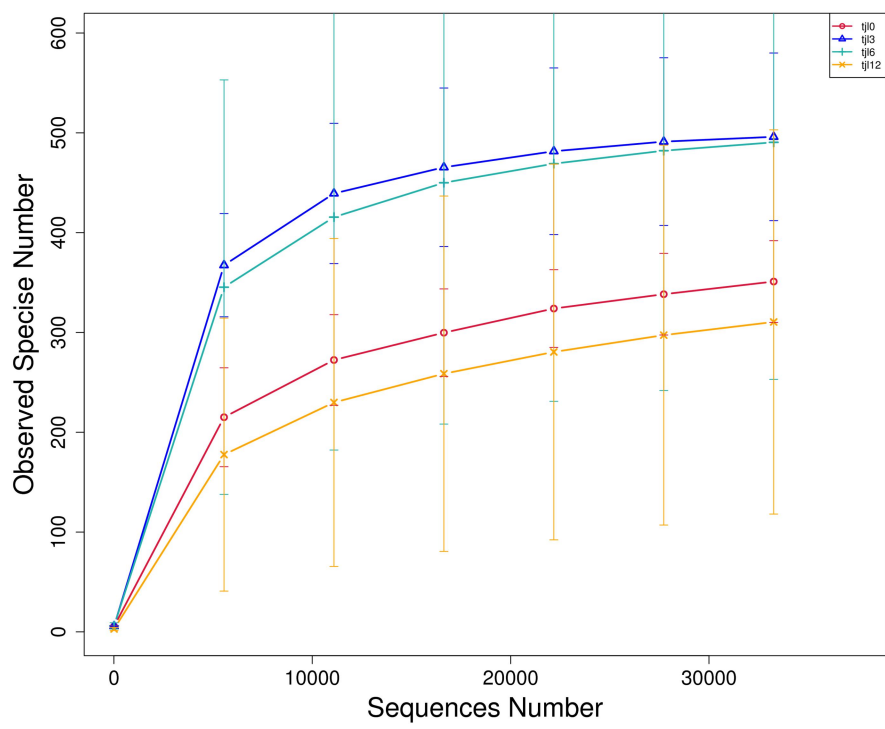

Suppl. Fig. 1

Supplement: S1 Fig — Tjl0, vitamin C-free supplemental basic diet group; tjl3, 300 mg vitamin C/kg basic diet group; tjl6, 600 mg vitamin C/kg basic diet group; tjl12, 1200 mg vitamin C/kg basic diet group. (PDF) [file pone.0300643.s001.pdf]
